# The Molecular and Mechanical Characteristics of Biomimetic Composite Dental Materials Composed of Nanocrystalline Hydroxyapatite and Light-Cured Adhesive

**DOI:** 10.3390/biomimetics7020035

**Published:** 2022-03-30

**Authors:** Pavel Seredin, Dmitry Goloshchapov, Vladimir Kashkarov, Yuri Ippolitov, Jitraporn Vongsvivut

**Affiliations:** 1 Solid State Physics and Nanostructures Department, Voronezh State University, University Sq. 1, 394018 Voronezh, Russia; goloshchapovdl@gmail.com (D.G.); kash@phys.vsu.ru (V.K.); 2 Scientific and Educational Center “Nanomaterials and Nanotechnologies”, Ural Federal University Named after the First President of Russia B. N. Yeltsin, Mir av., 620002 Yekaterinburg, Russia; 3 Department of Pediatric Dentistry with Orthodontia, Voronezh State Medical University, Studentcheskaya St. 11, 394006 Voronezh, Russia; dsvgma@mail.ru; 4 ANSTO—Australian Synchrotron, 800 Blackburn Road, Clayton, Melbourne, VIC 3168, Australia; jitrapov@ansto.gov.au

**Keywords:** biomimetics, nanodentology, nanocrystalline carbonate-substituted hydroxyapatite, bisphenol A-glycidyl methacrylate, degree of conversion, Vickers hardness

## Abstract

The application of biomimetic strategies and nanotechnologies (nanodentology) has led to numerous innovations and provided a considerable impetus by creating a new class of modern adhesion restoration materials, including different nanofillers. An analysis of the molecular properties of biomimetic adhesives was performed in this work to find the optimal composition that provides high polymerisation and mechanical hardness. Nanocrystalline carbonate-substituted calcium hydroxyapatite (nano-cHAp) was used as the filler of the light-cured adhesive Bis-GMA (bisphenol A-glycidyl methacrylate). The characteristics of this substance correspond to the apatite of human enamel and dentin, as well as to the biogenic source of calcium: avian eggshells. The introduction and distribution of nano-cHAp fillers in the adhesive matrix resulted in changes in chemical bonding, which were observed using Fourier transform infrared (FTIR) spectroscopy. As a result of the chemical bonding, the Vickers hardness (VH) and the degree of conversion under photopolymerisation of the nano-cHAp/Bis-GMA adhesive increased for the specified concentration of nanofiller. This result could contribute to the application of the developed biomimetic adhesives and the clinical success of restorations.

## 1. Introduction

One key problem in dental material science is the development of new restoration materials and the improvement of existing materials and their interactions with native dental tissue [1,2,3]. The clinical success of restoration involves the formation of a stable bond between the dental material and the native dental tissue due to the adhesive systems used [3,4,5,6,7]. The dental bonding system should be associated with the optimal ratio of the components [8], which provides the highest level of polymerisation, excellent adhesion and physical–mechanical properties. A non-optimal composition and a low degree of transformation reduce the clinical applicability of these systems.

Numerous innovations incorporating biomimetic strategies and nanotechnologies (such as nanodentology) provided considerable impetus in creating a new class of modern adhesion restoration materials [9]. It has repeatedly been shown that the trophic, mechanical (e.g., hardness and strength), physicochemical and performance properties of the adhesive polymer matrix can be enhanced by introducing various inorganic micro- and nanofillers [10,11,12]. The introduction of a nanofiller has been shown to result in the increased durability of dental restorations and improved bonding with dental tissues [10,13]. The search for new kinds of bioinspired fillers and ways to introduce them to the bond composition is an important scientific and practical task [2,14,15].

One filler often used for dental materials and bonds is calcium hydroxyapatite (HAp). The high efficiency of this compound is due to the similarity of its physicochemical characteristics with the inorganic component of human bone and dental tissue. Materials based on HAp have already been used to restore and regenerate tissue and modify cement and bonds [16,17,18]. Including HAp in dental adhesives changes their properties by increasing the degree of polymerisation, improving the adhesion and adhesive strength under macro-shear and enhancing the integration of the components with the native dental tissue [19,20]. However, the optimum content of nanocrystalline filler in the polymer matrix remains unknown.

The mechanical properties of a restoration material such as elastic modulus, material strength and the degree of polymerisation are important for clinical efficiency. A review of the literature has found a sufficient number of works [16,19,20,21] where the changes in the strength of adhesives filled with different nanofillers, including hydroxyapatite, were investigated. However, these reports lacked hardness values, estimates of the degree of polymerisation as a function of the nanofiller content and analysis of the molecular properties of the material.

Therefore, the main goal of our work was to determine the proper adaptive composition and molecular properties of a biomimetic adhesive based on bisphenol A-glycidyl methacrylate (Bis-GMA) filled with nanocrystalline carbonate-substituted hydroxyapatite (nano-cHAp), which provides a high degree of polymerisation and mechanical hardness.

## 2. Methods of Production and Study of the Samples

To obtain the biomimetic samples, we used bisphenol A-glycidyl methacrylate (Polysciences, Warrington, PA, USA, code 03344) commercial adhesive [8,22]. Nano-cHAp corresponding to the features of human tooth enamel and dentin [22,23,24] was applied as a filler for the light-cured Bis-GMA adhesive.

Samples of nano-cHAp were obtained using the wet chemistry method of titrating a concentrated solution of calcium hydroxide (Ca(OH)_2_) with 0.3 M orthophosphoric acid (H3PO4). The raw calcium hydroxide was obtained via the thermal annealing of chicken eggshells [22]. The morphological organisation of nano-cHAp synthesised according to this method is close to that of human hard dental tissues since it is formed of nanocrystals with the mean size of 20 × 20 × 50 nm [22]. This characteristic is an important feature for forming a biomimetic material capable of replacing the natural biogenic nano-cHAp. The nano-cHAp and the adhesive were mixed using an ultrasound homogeniser QSonica Q55 (Qsonica LLC, CT, USA) for 30 s.

To determine the adaptive composition and molecular properties of the biomimetic nano-filled adhesives, samples were prepared with different proportions of raw components (see Table 1). Ten samples of each type were prepared. After adding the nano-cHAp into Bis-GMA adhesive, the photopolymerisation process was performed with an ultraviolet diode illuminator (the width of the light band was 380–420 nm) for 60 s.

The molecular properties of the samples were investigated using Fourier transform infrared (FTIR) spectroscopy, including synchrotron FTIR microspectroscopy, at the Infrared Microspectroscopy beamline (Australian Synchrotron, Victoria, Australia). The experiments were performed with a Bruker VERTEX 80v spectrometer coupled with a Hyperion3000 FTIR microscope and liquid nitrogen-cooled narrow-band mercury cadmium telluride detector (Bruker Optik GmbH, Ettlingen, Germany). All FTIR absorption spectra were recorded using the attenuated total reflection (ATR) Diamond Platinum accessory (Bruker Optik GmbH, Ettlingen, Germany) within the spectral range of 3800–400 cm^−1^ at 4 cm^−1^ spectral resolution. Blackman–Harris 3-term anodisation, Mertz phase correction and a zero-filling factor of 2 were used as the default acquisition parameters in the OPUS software suite (v7.5, Bruker Optik GmbH, Ettlingen, Germany). Spectral data processing, baseline correction, averaging, determination of the peak positions and decomposition into components were performed in the Origin 8.0 program suite. Statistics was described using SigmaPlot.

After photopolymerisation, the microhardness of the synthesised biomimetic adhesive samples was determined using the Vickers technique and employing an optical microscope–hardness testing instrument (RMT-3, Moscow, Russia). Hardness values were averaged over ten measurements for each sample.

## 3. Results and Discussion

**Figure 1:**
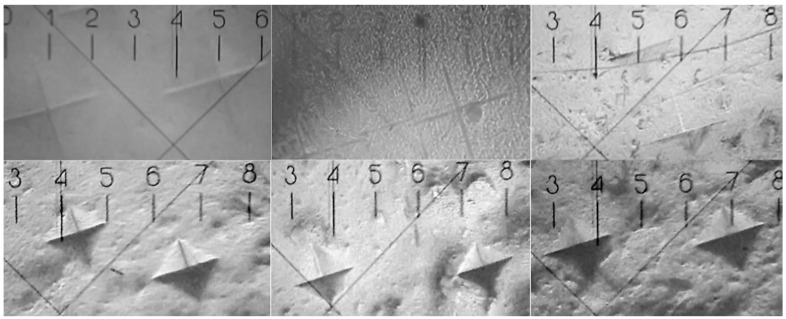
Determination of the microhardness of samples containing different proportions of nano-cHAp and adhesive from the indentation made by the diamond pyramid in the samples. Magnification ×130.

As noted previously, the microhardness of the samples is important to the clinical applicability of these materials in dental practice. The Vickers technique was used to measure the microhardness of the biomimetic Bis-GMA/nano-cHAp adhesives. Using this technique, microhardness can be determined based on the measurement of micro-indentations of a diamond pyramid pushed into the surface of a sample at a certain load (see Figure 1).

The microhardness number (HV) is determined from the following expression:

(1) Hμ=2Psin(θ2)d2

where *P* is the applied loading, *d* is the length of the indentation diagonal and *θ* = 136°, the angle at the top of the diamond square Vickers tip.

The microhardness was determined from at least five measurements at different points on each of ten specimens of a sample with specific proportions of the components. After that, the results were averaged and are presented in Table 1 as the mean ± standard deviation. The standard deviation did not exceed 3%.

From the data analysis in Table 1, the microhardness of the biomimetic Bis-GMA/nano-cHAp adhesive attains a maximum at the content of ~0.16 g nano-cHAp in 250 mL of Bis-GMA adhesive. After that, decreased microhardness is observed. The non-linear behaviour of the dependence of the microhardness values on the nano-cHAp content is due to the changes that occur in the molecular composition of the samples [16,19,20,21].

**Figure 2:**
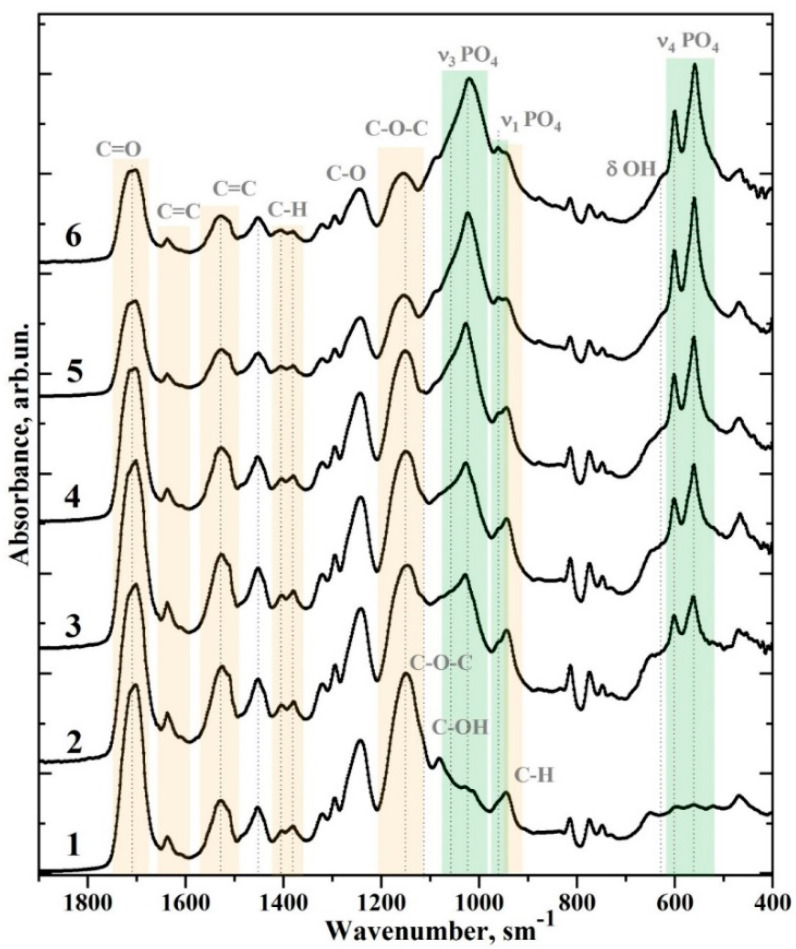
Comparison of FTIR spectra of dental biomimetic adhesives containing different amounts of nano-cHAp. (1) Sample #6 Bis-GMA/nano-cHAp (0.01 g); (2) sample #6 Bis-GMA/nano-cHAp (0.04 g); (3) sample #4 Bis-GMA/nano-cHAp (0.08 g); (4) sample #3 Bis-GMA/nano-cHAp (0.12 g); (5) sample #2 Bis-GMA/nano-cHAp (0.16 g); (6) sample #1 Bis-GMA/nano-cHAp (0.01 g).

**Figure 3:**
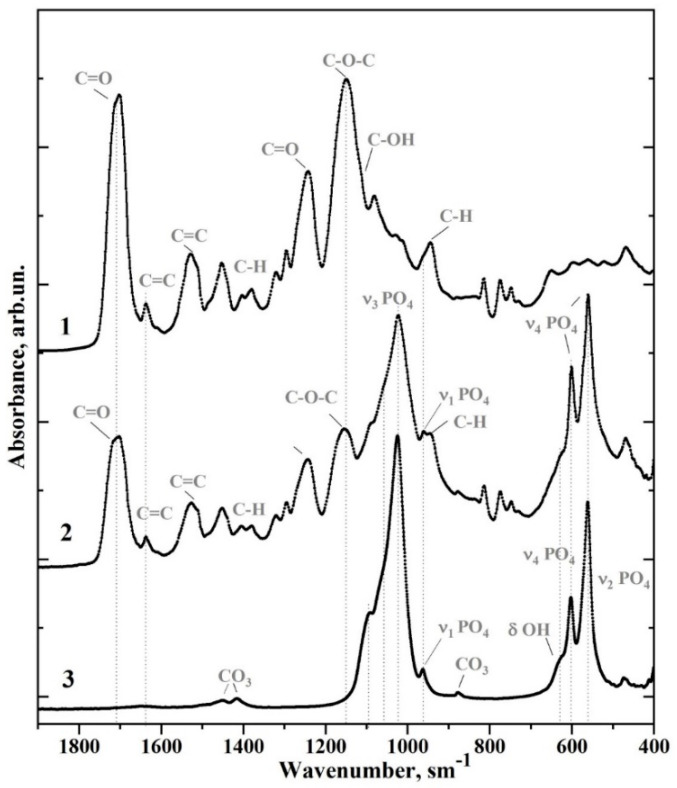
FTIR spectra of the (1) original Bis-GMA adhesive, (2) nanocrystalline carbonate-substituted calcium hydroxyapatite (nano-cHAp) and (3) sample #1 Bis-GMA/nano-cHAp (0.2 g).

In the case of a multi-component light-cured adhesive containing many active molecules and functional groups with the addition of nanofiller, the changes to the mechanical properties of the final material are as much due to the filler as to the original adhesive [25]. In order to analyse variations in the molecular structure of the adhesive system as a result of its modification by the use of the nano-cHAp filler, we used FTIR spectroscopy. Representative FTIR spectra for all types of biomimetic adhesive samples (see Table 1) are shown in Figure 2. Moreover, for more convenient analysis of the changes in molecular composition, the FTIR spectrum of the original Bis-GMA adhesive is presented in Figure 3, along with the spectrum of nano-cHAp and the spectrum of sample #1, biomimetic adhesive Bis-GMA/nano-cHAp (0.2 g), which contains the maximum amount of added nano-cHAp filler.

All of the FTIR spectra are presented in the range of 2000–400 cm^−1^. The most intense modes, associated with the characteristic vibrations of the original Bis-GMA adhesive and nano-cHAp, appear in this range. In order to compare these spectra, they were normalised to the maximum intensity. Preliminary consideration of the obtained experimental spectral data showed that the FTIR spectra of the samples involve the same set of vibrational modes associated with certain molecular bonds. Moreover, the intensity of the spectra of the same sample types differs insignificantly. Considering this fact, the spectra used for performing the following analysis are averaged over a certain type of sample.

The FTIR spectra (Figure 2 and Figure 3) were analysed after a literature search. Many HAp samples and various adhesive systems based on Bis-GMA [8,16,25,26,27] have been studied using molecular spectroscopy techniques. The frequencies of the active vibrations in the FTIR spectra and their association with certain molecular groups and ions are presented in Table 2. Comparison of the reference and experimental data showed that the main and most intense modes in the FTIR spectra for all of the samples (see Table 2) could be associated with either the characteristic vibrations of nano-cHAp [28,29] or the vibrations of the adhesive Bis-GMA [8,25,26].

In the range of 1110–960 cm^−1^, the most intensive band in the FTIR spectra assigned to nano-cHAp occurred and was associated with the vibrations of υ_1_ and υ_3_ PO_4_^3−^ [21,22,27]. The second most intense vibrational band was associated with nano-cHAp filler (two separate peaks in the range of 600–550 cm^−1^), specifically, the υ_4_ PO_4_^3−^ mode [21,22,27]. Moreover, a low-intensity doublet in the spectra of the samples was observed in the range of 1451–1414 cm^−1^, which can be associated with the symmetric and asymmetric vibrations, υ_3_, of the CO_3_^2−^ groups in nano-cHAp, when a carbonate anion replaces the PO_4_ group in apatite [30].

**Figure 4:**
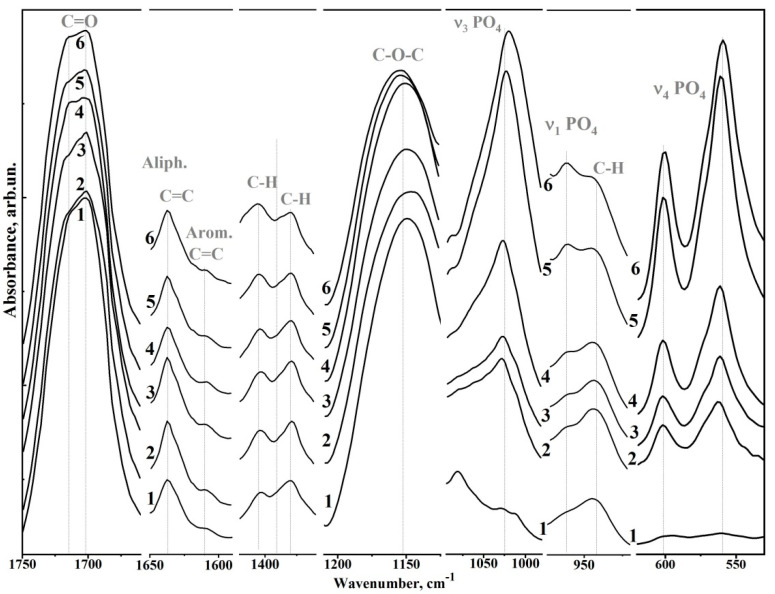
Spectral features of biomimetic adhesives. (1) Sample #6 Bis-GMA/nano-cHAp (0.01 g); (2) sample #6 Bis-GMA/nano-cHAp (0.04 g); (3) sample #4 Bis-GMA/nano-cHAp (0.08 g); (4) sample #3 Bis-GMA/nano-cHAp (0.12 g); (5) sample #2 Bis-GMA/nano-cHAp (0.16 g); (6) sample #1 Bis-GMA/nano-cHAp (0.01 g).

The characteristic vibrations associated with the Bis-GMA adhesive are an absorption band at 1750–1665 cm^−1^ associated with C=O stretching in the methacrylate group; a band at 1150–1120 cm^−1^ associated with C–O–C stretching; a triplet in the range of 1320–1120 cm^−1^ related to the C–O bond vibration; a band at 1528 cm^−1^ associated with the aromatic C=C group; and a band at 1450 cm^−1^ associated with C–H bending vibrations [8,25,26]. In addition, in the FTIR spectrum of unaltered Bis-GMA adhesive (see Figure 3), several low-intensity absorption bands can be attributed to specific molecular groups (see Table 2). More detailed spectral features for seven fingerprint regions containing the main characteristic vibrations of nano-cHAp and Bis-GMA are depicted in Figure 4.

In Figure 4 (from high to low wavenumbers), the following regions are presented: (1) the range of 1750–1675 cm^−1^, where C=O vibrations of the ether group (–COOCH_3_), a component of the Bis-GMA adhesive, appear (the band has a fine structure of an unresolved doublet); (2) the range of 1650–1590 cm^−1^, where aliphatic C=C and aromatic C–C vibrations of Bis-GMA are present; (3) the region of 1410–1375 cm^−1^, where the C–H bending doublet of the methacrylate group I Bis-GMA occurs; (4) the region of 1275–1125 cm^−1^, where the aromatic C–O and C–O–C stretches of Bis-GMA are located; (5) a spectral band in the range of 1100–980 cm^−1^ is associated with the asymmetric O–P–O stretching mode υ_3_ of PO_4_^3−^; (6) the region of 975–925 cm^−1^, where the valence mode, υ_1_, of the P–O bond (υ_1_ of the PO_4_^3−^) occurs as well as the C–H vibrations of Bis-GMA; and (7) the range of 610–530 cm^−1^, where the doublet υ_4_ (O–P–O bending mode) of PO_4_^3−^ is located.

Analysis of Figure 2 shows that the intensities of the active vibrations in the FTIR spectra change predictably depending on the ratio of the components of the biomimetic Bis-GMA/nano-cHAp adhesive, thus confirming the changes in the composition of a material. The following spectral features are affected by changes in the amount of nano-cHAp filler added.

For example, vibrations associated with the C=O group in ether (–COOCH_3_) shifted towards the 1717 cm^−1^, and the simultaneous redistribution of intensities between the components in the unresolved doublet was observed. The intensities of the nano-cHAp peaks in the high-frequency range (i.e., the shoulder) became noticeably higher. In addition, a noticeable redistribution of the peaks of the C–H bending doublet (1410–1375 cm^−1^) and the υ_4_ PO_4_^3−^ doublet (610–530 cm^−1^) was observed. Moreover, with the increased addition of nano-cHAp filler, the intensity of the υ_1_ and υ_3_ modes (PO_4_^3−^) increased, and the position of the υ_3_ (PO_4_^3−^) peak noticeably shifted from 1027 cm^−1^ to 1020 cm^−1^. The behaviour of the aromatic C–O (1243 cm^−1^) and C–O–C stretch (1150 cm^−1^) vibrations also indicated molecular changes. With increased nano-cHAp content, the aromatic C–O band did not change, while the vibration band associated with the C–O–C bond shifted towards higher frequencies.

The degree of conversion for the adhesive material can be determined using the FTIR data [31]. To accomplish this, the ratio of integral intensities for the bands related to the aliphatic (C–C) bonds versus the bands related to the aromatic (C=C) bonds before and after polymerisation must be determined. The aliphatic C=C bond contribution was determined from the intensity of the vibrations near 1637 cm^−1^, while the contribution of the aromatic (C=C) bonds was determined from the intensity of the vibrations near 1610 cm^−1^. Calculations were performed for ten samples of pure Bis-GMA adhesive and each of the ten biomimetic adhesive specimens. After that, the mean values for the degree of conversion for each group of the specimens were determined, and the standard deviation was calculated, which did not exceed 2% (see Table 1).

Calculating the degrees of conversion for the samples showed that the original adhesive based on Bis-GMA contained 22.0% ± 1.4% non-polymerised bonds, which agreed with the calculations presented for the adhesive based on Bis-GMA/HEMA from [31]. When nano-cHAp was added, the degree of conversion (polymerisation) increased, attained its maximum at ~93%, then decreased.

**Figure 5:**
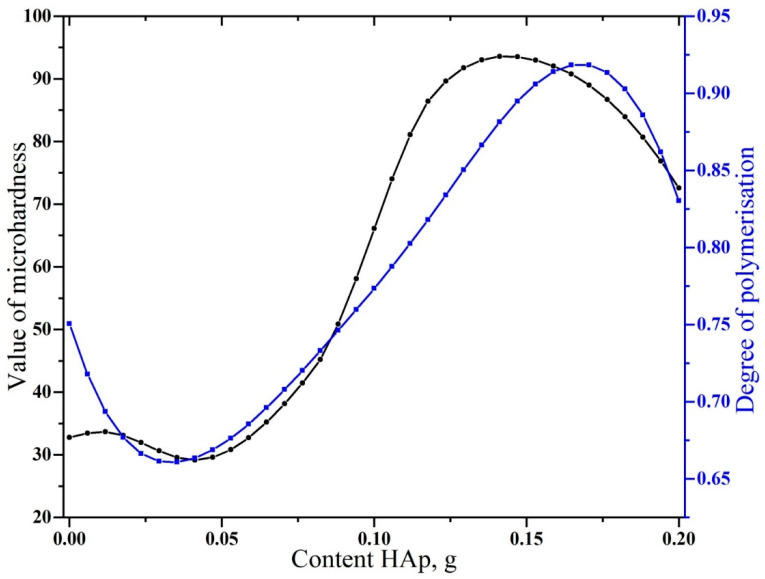
Comparison of the dependence of microhardness, *H_μ_* (VH), and degree of conversion of biomimetic adhesives on nanofiller admixture.

Spline curves of the dependence of the microhardness and degree of polymerisation of the samples on the amount of nano-cHAp added to the biomimetic adhesive are presented in Figure 5. Both values, representing the mechanical and molecular properties of the synthesised biomimetic adhesives, depended similarly on the filler content. Simultaneous graphical analysis allowed the determination of the range of optimal compositions for the biomimetic adhesive, which provided a maximal value of the microhardness and the degree of conversion during polymerisation. From the calculations, it followed (see Figure 5) that the content of nano-cHAp with characteristic morphological characteristics (20 × 20 × 50 nm) should be between 0.125 and 0.135 g per 250 mL of Bis-GMA (Figure 5).

Analysing the results of our investigations and comparing them with the data obtained from similar experiments [32,33,34], it is possible to make the following conclusions. The molecular properties of the synthesised biomimetic adhesives were inherited from the original Bis-GMA adhesive and the nano-cHAp used for their production. They are also due to the proportions of these components in the final adhesive composition.

The redistribution of the intensities observed in the FTIR spectra, as well as the change in the arrangement of the bands related to both Bis-GMA and nano-cHAp, indicated the interaction of the surface bonds in the nanofiller with the active molecular groups of the adhesive. The mechanisms of such interaction were previously considered in [27,32,33,34]. The formation of additional bonds due to the large specific surface area of the filler nanoparticles results in the change in characteristics and is referred to as the molecular structure of an adhesive and a filler. The change in the intensities of the vibrational modes of C=C, C–O, C–O–C and υ_4_ PO_4_^3−^ (O–P–O bending mode) occurred simultaneously. Moreover, as previously shown, the width of the high-frequency maximum of υ_4_ PO_4_^3−^ of the doublet (near 605 cm^−1^) was correlated with the changes in the local and spatial structure of bioapatite [27]. The infrared splitting factor (IRSF), used to estimate bioapatite crystallinity, is the sum of the peak intensities at 605 and 562 cm^−1^ (υ_4_ PO_4_^3−^ vibrational mode) divided by the intensity of the valley between them. Calculations showed that the IRSF had a minimum in the range of compositions of 0.12–0.16 g and a characteristic value for fresh bone and dentin [27].

The transformation of the molecular composition was also observed as the change in strength (hardness) of the Bis-GMA/nano-cHAp adhesive, as a greater degree of conversion under photopolymerisation decreases the probability of degradation and polymer plasticisation [21].

Before this work, it had been shown that the involvement of nanoparticles improved the mechanical properties of dental composites. The addition of the silicon nanoparticle fillers to adhesion systems improved the mechanical properties and elastic modulus by changing the distribution of stresses caused by polymerisation [32]. The modification of adhesive systems based on Bis-GMA, TEGDMA and HEMA with the use of HAp nanocrystallites from the work of Leitune et al. [21] correlated well with the dependence of the mechanical properties of adhesives on increased added nanofillers observed in this work. Using HAp nanoparticles with a mean size of ~27 nm, enhanced microhardness values were attained in the range of ~32.35 MPa. After that, further increasing the HAp nanoparticle content in the adhesive led to a characteristic decay of mechanical properties. However, in this work, even greater microhardness values (HV) with an increased degree of conversion exceeding 63.84% were obtained. The latter was attained by Leitune et al. [21] for the addition of 1% HAp by mass.

According to previous work, the values of VH for natural enamel and dentin are within the limits of ~270–360 HV and 50–60 HV, respectively [35,36]. From the results obtained in this study (Figure 5), by modifying Bis-GMA with nano-cHAp, it is possible to attain a value of VH greater than the dentin hardness but less than that of dental enamel. This composite would probably redistribute natural loads between the anatomic tissues efficiently.

The practical results of our studies have already been demonstrated in our previous works [37,38], where the modification of Bis-GMA adhesive with nano-cHAp made it possible to form a biomimetic hybrid interface integrated with the structure of the tissue. This result showed that the addition of HAp into the adhesive system could prevent degradation of the hybrid layer and remain efficiently bound with time [19,20,21].

It has been repeatedly noted that an important factor of the process is the size of the nanofiller particles. Large particles can lead to the agglomeration of the particles and the degradation of the mechanical properties of the interface [34]. However, the type of nanofiller also has a great impact on the final properties of the modified material. Unlike many previous similar investigations where HAp nanoparticles and other inorganic nanomaterials were applied as the filling of adhesion systems, this work used nanocrystals of cHAp with the mean size of 20 × 20 × 50 nm, obtained according to our new method. These nanocrystals are characteristic of the native dental tissue. The uniform distribution of the nano-cHAp filler in the adhesive matrix, as well the interaction with the molecular groups of the filler, favours the changes in molecular bonds that were confirmed using FTIR spectroscopy; as a result, the mechanical characteristics of the material were considerably improved.

## 4. Conclusions

With the use of light-cured Bis-GMA adhesive and nano-cHAp corresponding to an aggregate set of characteristics of the apatite of human enamel and dentin obtained from avian eggshells, a biogenic source of calcium, biomimetic Bis-GMA/nano-cHAp adhesives were synthesised. The introduction and distribution of the nano-cHAp filler in the adhesive matrix, as well as its interaction with molecular groups of the adhesive, resulted in changes to the chemical bonds that were confirmed via FTIR spectroscopy. In summary, for the specified nanofiller concentration, increased values of the VH and degree of conversion were attained simultaneously in the light-cured Bis-GMA/nano-cHAp adhesive. This result will considerably influence the application of the developed biomimetic adhesives and the clinical success of tooth restoration using these composites.

## Figures and Tables

**Table 1:** Composition of the synthesised biomimetic adhesive samples.

Sample	Bis-GMA, mL	nano-cHAp, g	*H_μ_* (HV)	Degree of Conversion
#1	250	0.2	33.68	0.827 ± 0.012
#2	250	0.16	29.16	0.93 ± 0.016
#3	250	0.12	43.56	0.80 ± 0.014
#4	250	0.08	87.90	0.74 ± 0.015
#5	250	0.04	91.82	0.68 ± 0.015
#6	250	0.01	72.60	0.654 ± 0.016

**Table 2:** Molecular vibrations in the FTIR spectra of biomimetic adhesives.

Wavenumber (cm^−1^)	Assignment	Compound	References
1750–1665	C=O stretch, (–COOCH_3_) ether	Bis-GMA*	[8,25,26]
1637	Aliphatic C=C	Bis-GMA	[8,25,26]
1610	Aromatic C=C	Bis-GMA	[8,25,26]
1528, 1510	Aromatic C=C	Bis-GMA	[8,25,26]
1451	C–H bending, υ_3_ CO_3_^2−^ in HAp lattice	Bis-GMA, nano-cHAp*	[22]
1414	υ_3_ CO_3_^2−^ in HAp lattice	nano-cHAp	[8,25,26]
1403, 1380	C–H bending	Bis-GMA	[8,25,26]
1320, 1295	C–O stretch doublet	Bis-GMA	[8,25,26]
1243	Aromatic C–O	Bis-GMA	[8,25,26]
1150	C–O–C stretch	Bis-GMA	[8,25,26]
1120	C–O–C stretch	Bis-GMA	[8,25,26]
1090	υ_3_ PO_4_^3−^	nano-cHAp	[22]
1081	C–OH stretch	Bis-GMA	[8,25,26]
962	υ_1_ PO_4_^3−^ (stretching mode of the P–O bond)	nano-cHAp	[22]
960, 945	C-H	Bis-GMA	[8,25,26]
878, 870	CO_3_^2−^ in HAP lattice	nano-cHAp	[22]
815	C–C–O stretch	Bis-GMA	[8,25,26]
630	δ OH	nano-cHAp	[22]
602, 597	υ_4_ PO_4_^3−^ O–P–O bending modes	nano-cHAp	[22]
562.560		nano-cHAp	[22]

Bis-GMA*—bisphenol A-glycidyl methacrylate; nano-cHAp*—nanocrystalline carbonate-substituted calcium hydroxyapatite; HAp lattice—hydroxyapatite crystal lattice.

## Data Availability

The data that support the findings of this study are available from the corresponding author upon reasonable request.
